# Survey to determine the farm-level impact of Schmallenberg virus during the 2016–2017 United Kingdom lambing season

**DOI:** 10.1136/vr.104866

**Published:** 2018-09-29

**Authors:** Jessica Eleanor Stokes, Rachael Eugenie Tarlinton, Fiona Lovatt, Matthew Baylis, Amanda Carson, Jennifer Sarah Duncan

**Affiliations:** 1 Institute of Infection and Global Health, University of Liverpool, Liverpool, UK; 2 School of Veterinary Medicine and Science, University of Nottingham, Nottingham, UK; 3 Health Protection Research Unit in Emerging and Zoonotic Infections, University of Liverpool, Liverpool, UK; 4 Surveillance Intelligence Unit, Animal and Plant Health Agency, Surrey, UK; 5 Institute of Veterinary Science, University of Liverpool, Liverpool, UK

**Keywords:** schmallenberg virus, surveys, disease impact, ewes, lambs

## Abstract

Schmallenberg virus (SBV) causes abortions, stillbirths and fetal malformations in naïve ruminants. The impact of the initial outbreak (2011/2012) on British sheep farms has been previously investigated, with higher farmer perceived impacts and increased lamb and ewe mortality reported on SBV-affected farms. After several years of low, or no, circulation the UK sheep flock once again became vulnerable to SBV infection. Re-emergence was confirmed in autumn 2016. This study reports the analysis of a questionnaire designed to determine the farm-level impact of SBV on the 2016/2017 UK lambing period. Higher neonatal lamb mortality, dystocia and associated ewe deaths, and higher perceived impacts on sheep welfare, flock financial performance and farmer emotional wellness were reported on SBV confirmed (n=59) and SBV suspected (n=82), than SBV not suspected (n=74) farms. Additionally, although few farmers (20.4 per cent) reported previously vaccinating against SBV, the majority (78.3 per cent) stated they would vaccinate if purchasing at less than £1 per dose. These results are largely comparable to the findings reported for the 2011/2012 outbreak, highlighting the ongoing impact of SBV on sheep farms. If SBV continues to re-emerge cyclically, the economic and animal welfare costs to the UK sheep farming industry will continue.

## Introduction

Schmallenberg virus (SBV) is a teratogenic virus, transmitted by *Culicoides* biting midges, that infects ruminants.^1–3^ The first reports of SBV came from Germany and the Netherlands in autumn 2011 where cattle presenting with diarrhoea, febrile episodes and a reduced milk yield tested negative for all known bovine pathogens. Following this initial description, reports of SBV quickly emerged throughout Europe, with transmission facilitated by the dispersal of the *Culicoides* vectors by wind and a completely naïve host population.^4^

Infections of adult ruminants are typically asymptomatic, or present with only mild clinical signs, as observed in cattle.^5–7^ However, if a naïve animal is infected for the first time during the vulnerable period of gestation, infection can result in stillbirths and fetal abnormalities, including arthrogryposis and hydranencephaly.^8^ Infection early in pregnancy has also been linked to lower conception rates, abortions and reduction in weaning rates.^9–12^ These associated clinical signs of disease are particularly problematic for block breeding production systems, with high reported losses from the disease in early lambing sheep flocks in 2011/2012.^10 13–15^

Farm-level disease incidence is known to vary significantly, as does the resulting impact. The UK Animal and Plant Health Agency (APHA) found 6 per cent of farmers from SBV confirmed or SBV suspected farms were less likely to farm sheep again the next year, compared with only 1.8 per cent of farmers from farms unaffected by SBV in the initial outbreak.^16^ Economic costs may also be higher than originally considered due to the difficulties in quantifying certain types of losses. For example, higher barren rates and reduced fertility are reported in some studies.^10 12 17^ Furthermore, due to the associated deformities, dystocia is relatively common, potentially resulting in additional losses of ewes while birthing malformed lambs.^3^ Critically, all studies estimating the impact of SBV have acknowledged the issue of under-reporting; SBV is not a listed notifiable disease by the World Organisation for Animal Health, with farmers from many member states of the EU voluntarily submitting samples and paying for confirmation testing and therefore accurate estimates of the true impact of disease are hard to establish.^14 18^

The unpredictable and intermittent nature of SBV has impacted on farmer uptake of vaccination; the main effective control measure. Having circulated and successfully overwintered between the 2011/2012 and 2012/2013 lambing seasons, SBV reports in the UK reduced dramatically in 2014. Several studies in Europe described very low circulation between 2014 and 2015.^19–21^ These studies highlighted large SBV-naïve populations vulnerable to reinfection, particularly as time progressed and vaccinations became no longer available.

After three years of low SBV circulation SBV re-emerged in Europe; by December 2016 deformed lambs were confirmed positive for SBV in the UK.^22^ With the vaccines withdrawn from the market due to poor uptake, and the duration of natural immunity unknown, the UK national flock was likely to be susceptible to infection.

This study aimed to measure and compare the impact of the 2016/2017 re-emergence of SBV on sheep flocks in the UK with the impact reported during the initial 2011/2012 outbreak. Expanding on a study following the initial outbreak,^16^ a questionnaire was designed to determine the impact of SBV during the 2016/2017 lambing period on lamb and ewe losses, farmer perceived emotional, financial and welfare costs and views on vaccination.^16^

## Materials and methods

To directly compare the impact of SBV on the 2016/2017 lambing season with that reported in 2011/2012 a questionnaire was designed to closely match that of Harris and others.^16^ Additional questions were designed by the authors. The questionnaire was tested by four sheep farmers and feedback incorporated into the final questionnaire (online supplementary information). Questionnaire participation was voluntary and open to all UK sheep farmers. The final version was launched online on March 24, 2017 using SurveyMonkey (California, USA). The online questionnaire was publicised periodically through Twitter, with support from AHDB Beef and Lamb, Sheep Veterinary Society and the APHA. A link to the online questionnaire was also handed out by veterinary students from both universities while on Easter lambing placements. A further 250 questionnaires were sent out by the APHA to farmers who had submitted samples for SBV testing in England and Wales on June 1, 2017.

A total of 32 questions were asked to determine farm demographics, lambing productivity and mortality, ewe mortality, vaccination history, the farms’ SBV status and farmers’ perception towards the impact of SBV on flock welfare, financial performance and the farmers’ own emotional wellbeing. The farms’ SBV status was determined by responses to two questions within the questionnaire and coauthors’ opinions of additional comments. The categories were:

SBV confirmed: Farms where a suspected lamb was confirmed positive for SBV by laboratory testing (at the time of the study the APHA were offering the RT-PCR testing of cerebral cortex or fresh brainstem from lambs).

SBV suspected: SBV was suspected by the farmer or their veterinarian. This includes farms that had positive testing of ewe blood samples for ELISA serology, and those that had lambs sent off for laboratory testing (with relevant clinical signs) that were not confirmed positive.

SBV not suspected (by respondent): No report of suspected SBV (based on a lack of farmer observed clinical signs) and did not send off any samples for SBV testing.

### Data analysis

All online results were downloaded from SurveyMonkey on June 19, 2017. All paper versions were manually entered to create a master copy. Responses were checked for consistency, any insufficiently completed responses were removed from the working copy.

### Mortality definitions

To allow direct comparison to the previous 2011/2012 study^16^ the same calculations and definitions were applied, briefly:

Lamb mortality (%)=100*(lambs dead from any cause within one week/total lambs born)

Lambing mortality (%)=100*(lambs dead from any cause within one week/non-barren ewes)

Ewe mortality (%)=100*(number of ewes that died during lambing/non-barren ewes)

Responses for farm demography, lambing productivity, lamb mortality, ewe mortality and the farmers’ impact perception questions were compared across SBV categories. All maps were created in QGIS V.2.2.0 and all statistical analyses were completed in R, V.3.4.1.^23 24^ Analysis of variance (ANOVA) and Tukey’s honestly significant difference post hoc tests were used to compare differences across the SBV categories for continuous data. If the Levene’s test for homogeneity of variance was significant the alternative Welch test was used with a Games-Howell post hoc test. For categorical data and vaccination history, Pearson’s χ^2^ tests were completed. Where the assumptions of the χ^2^ were violated a Fisher’s exact test was used.

### Malformation definitions

Farmers were asked to describe any malformations seen in lambs on farm, regardless of SBV status. These descriptions were then coded separately by the authors (JES, RET and JSD) into five groups previously described^16^ and ‘Other’. Themes resulting from the ‘Other’ group created two more groups: fused joints and eye-related deformities. The coding was undertaken blind and SBV category masked to reduce the possibility of bias. The coded results were then combined; those that did not match exactly (n=33) were reviewed and a consensus reached between authors.

## Results

In total, 318 respondents participated in the survey, 232 online and 86 via post (postal response rate 34.4 per cent). All 86 postal responses were included in the survey; however, only 129 of the online responses were completed in sufficient detail to be included, leaving 215 useable responses. Not all participants answered every question.

### Farm demographics

**Figure 1:**
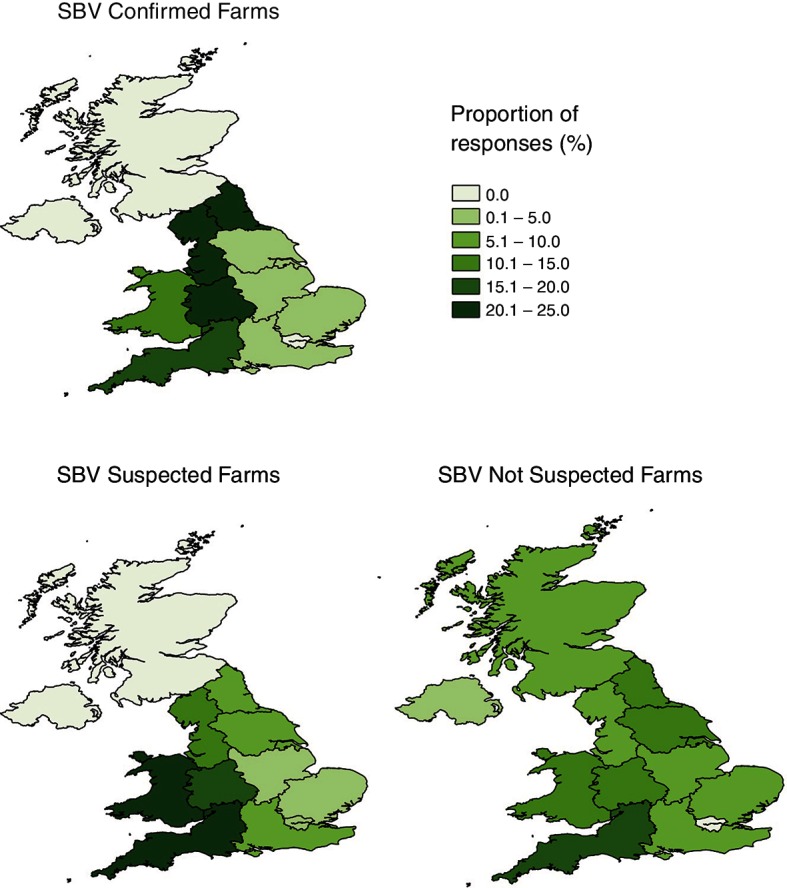
Proportion of responses by region for each of the SBV categories. Total responses: SBV confirmed farms (n=59), SBV suspected farms (n=82), SBV not suspected farms (n=74). SBV, Schmallenberg virus.

The majority of respondents were from the west of England and Wales (n=214, 65.0 per cent). In total, 27.4 per cent of respondents were from SBV confirmed farms, 38.1 per cent from SBV suspected farms and 34.4 per cent from SBV not suspected farms (n=215) (figure 1). Flock types did not significantly differ in SBV categories (P=0.17, table 1) with a total of 56.5 per cent of respondents defining their flock as crossbreeds/commercial animals. There was a non-significant trend towards a difference between the SBV categories of the two farm types (P=0.07, table 1): specifically, there was a greater proportion of upland/hill farms in the SBV not suspected category than lowland farms.

**Table 1:** Farm type and flock type by SBV category

Description (n)	SBV confirmed n=59 (%)	SBV suspected n=82 (%)	SBV not suspected n=74 (%)	P values
Farm type (214)*				0.07
Lowland (164)	47 (79.7)	67 (82.7)	50 (67.6)	
Upland/hill (50)	12 (20.3)	14 (17.3)	24 (32.4)	
Flock type (214)*				0.17
Crossbreeds/commercials (121)	39 (66.1)	45 (55.6)	37 (50.0)	
Pedigree/pure bred (93)	20 (33.9)	36 (44.4)	37 (50.0)	

*Farmers had to select one option to describe their flock. Not all farmers answered every question. Percentages may not equal to 100 due to rounding.

SBV, Schmallenberg virus.

### Breeding seasons, scanning rates and lambing percentages

The earliest reported date for the ram to be put in with the ewes was the May 18, 2016; the latest date of ram removal was April 16, 2017. The reported duration of the mating season was similar, but slightly shorter for SBV not suspected farms when compared with confirmed and suspected farms (table 2).

**Table 2:** Farm breeding demographics by SBV category

Summary description	SBV confirmed (n=59)	SBV suspected (n=82)	SBV not suspected (n=74)	P values
**Duration of mating season**				0.09
Responses (n)	58	79	69	
Earliest start date	June 10, 2016	May 18, 2016	July 28, 2016	
Latest end date	February 2, 2017	April 1, 2017	April 16, 2017	
Season duration				
Median (days)	77	61	56	
Min (days)	15	14	21	
Max (days)	174	264	148	
IQR	50.3–96.5	42.0–88.5	41.0–84.0	
**Duration of lambing season**				<0.001*
Responses (n)	58	79	64	
Earliest start date	October 30, 2016	October 10, 2016	January 3, 2017	
Latest end date	June 2, 2017	June 4, 2017	June 30, 2017	
Season duration				
Median (days)	64.5	52.0	40.0	
Min (days)	9	6	5	
Max (days)	161	153	115	
IQR	38.3–88.8	40.0–81.0	26.8–57.3	
**Tupped ewes that were barren (%)**				0.561
Responses (n)	48	57	38	
Median	3.7	4.3	3.2	
Min	0	0	0	
Max	27.3	35.2	66.7	
IQR	1.9–6.3	2.7–7.3	1.9–5.1	
**Lambing percentage**				0.725
Responses (n)	59	72	63	
Median	174.3	173.0	166.7	
Min	100.0	110.2	50.0	
Max	212.4	242.9	264.4	
IQR	157.6–185.0	152.3–185.9	146.2–185.6	
**Scanning percentage**				0.750
Responses (n)	50	58	41	
Median	175.0	172.5	176.0	
Min	118.0	100.0	100.0	
Max	223.0	214.0	250.0	
IQR	160.0–188.0	159.3–187.0	160.0–187	

*Analyses of variance (ANOVA) were conducted except where Levene’s test determined non-homogeneity of variance where instead the alternative Welch ANOVA was conducted.

SBV, Schmallenberg virus.

**Figure 2:**
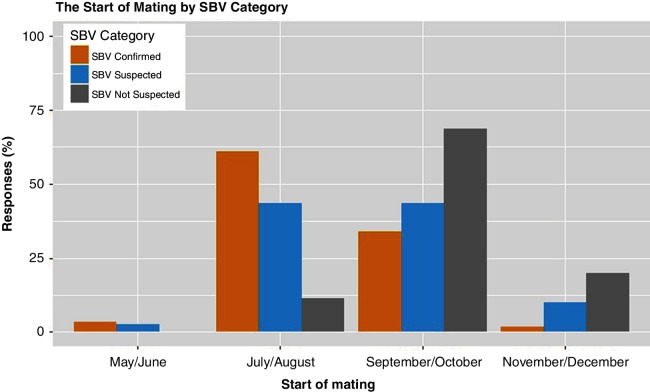
The reported start of mating by SBV category. Fisher’s exact test P<0.001. SBV, Schmallenberg virus.

The start dates for mating were grouped into four categories: ‘May/June’ (spring/early summer), ‘July/August’ (mid-summer), ‘September/October’ (early autumn) and ‘November/December’ (late autumn/winter) to allow comparison by SBV category for different seasonal mating strategies. There was a significant difference between the mating start dates on SBV confirmed and SBV suspected farms compared with SBV not suspected farms (P<0.001; post hoc test with Bonferroni’s correction P<0.001) with earlier mating start dates reported on SBV confirmed and SBV suspected farms (figure 2).

The median duration of lambing season was significantly different across the categories, with SBV not suspected farms recording a median lambing duration of 24.5 days less than SBV confirmed farms (P<0.001, table 2).

There was no significant difference between the SBV categories and barren rates, scanning percentages or lambing percentages (table 2).

### Lamb mortality

Significantly higher lamb mortality was observed on SBV confirmed farms (median of 9.1 lamb deaths per 100 born) and SBV suspected farms (median of 7.6 per cent) than on SBV not suspected farms (median of 5.7 per cent) (P*<*0.001, table 3).

**Table 3:** Lamb mortality and lambing mortality by SBV category

Summary	SBV confirmed (n=59)	SBV suspected (n=82)	SBV not suspected (n=74)	P values
**Lamb mortality (per lambs born)**				<0.001
Responses (n)	56	70	50	
Median	9.1	7.6	5.7	
Min	0	0	0	
Max	63.4	47.4	28.6	
IQR	6.8–15.2	4.5–13.1	1.5–9.1	
**Lambing mortality (per pregnant ewes)**				<0.001
Responses (n)	56	70	50	
Median	15.2	12.7	8.4	
Min	0	0	0	
Max	126.8	100.0	53.3	
IQR	10.9–24.8	8.1–20.7	2.3–15.2	

Tukey’s honestly significant difference (HSD) was performed for all significant analyses of variance (ANOVA) to determine the observable difference.

SBV, Schmallenberg virus.

Lambing mortality was also significantly higher on SBV confirmed farms (median of 15.2 lamb deaths per 100 pregnant ewes) than SBV suspected (median 12.7 per cent) or SBV not suspected farms (median 8.4 per cent) (P<0.001, table 3).

**Figure 3:**
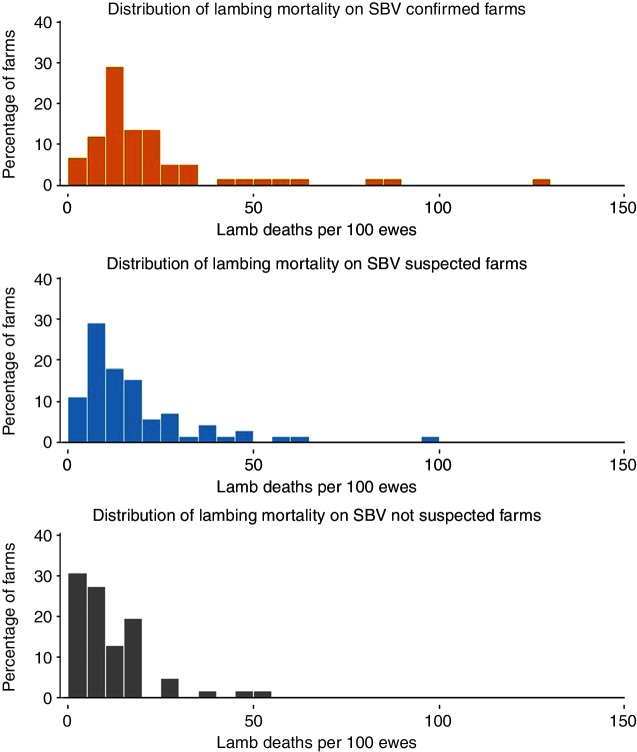
Distribution of lambing mortality (per cent) (lamb deaths per 100 ewes) by SBV category. SBV, Schmallenberg virus.

Particularly high lambing mortality (more than 40 per cent lambing mortality) was observed more frequently on SBV confirmed farms (13.8 per cent) and SBV suspected farms (8.3 per cent) than on SBV not suspected farms (3.2 per cent). Far more outliers were observed for SBV confirmed farms than SBV not suspected farms (figure 3).

### Abnormalities in lambs

**Figure 4:**
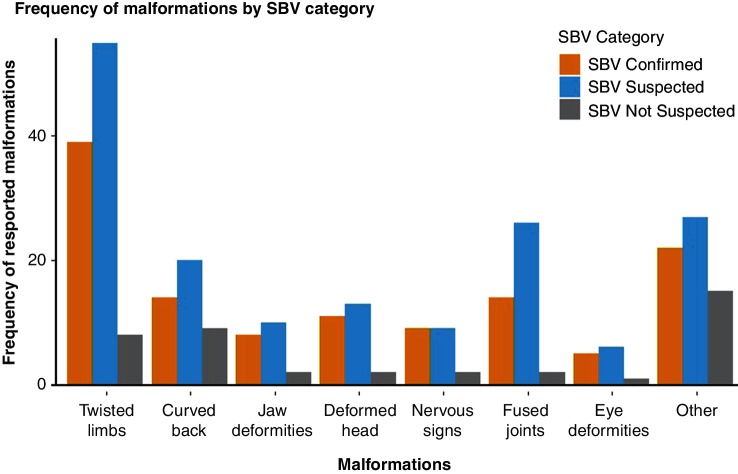
The farm-level frequency of reported malformations by SBV category. As farmers may have described a lamb as having multiple malformations (ie, ‘twisted limbs and an undershot jaw’) the frequencies do not sum to the total number of farmers describing malformations. Not all farmers answered all questions. Under ‘other’ the following abnormalities were reported by the farmers: for SBV confirmed: weak (4), small lamb (2), no muscle on back (2), missing ears (2), long legs (1), stillborn ‘rotten’ (1), cyst on head (1), large testicles (1); for SBV suspected: long legs (3), weak (3), no bone structure (3), cyst on head (2), internally deformed (2), two heads (1), protruding spine (1), short legs (1), small lamb (1), stillborn ‘rotten’ (1), missing ears (1); for SBV not suspected: stiff neck (2), long legs (1), small lamb (1), thin legs (1), internal organs external (1), conjoined (1). SBV, Schmallenberg virus.

A greater number of malformations were reported by SBV suspected than SBV confirmed or SBV not suspected farms. Twisted limbs were the most frequently reported malformation on SBV confirmed and SBV suspected farms, a curved back was the most frequently reported malformation on SBV not suspected farms (figure 4). The most common reported eye deformities on SBV confirmed and suspected farms (n=9) were a lack of eyes (three and two reports, respectively) and blindness (one and two reports, respectively). One farmer who did not suspect SBV on farm also reported a lack of eyes on one lamb.

At least one malformation was described by 84.7 per cent (50/59) of SBV confirmed farms, 87.8 per cent (72/82) of SBV suspected farms and 31.1 per cent (23/74) of SBV not suspected farms.

### Ewe losses

Ewe mortality during the lambing period was not significantly different across the SBV categories; however, the number of ewes that died while giving birth to a deformed lamb was significantly different between the groups (P=0.011). In total, 30.9 per cent (n=17) respondents from SBV confirmed farms reported one or more ewe deaths due to birthing a malformed lamb, similarly 26.4 per cent (n=19) reported the same for SBV suspected farms, while only 5.6 per cent (n=3) of respondents on SBV not suspected farms reported any ewe deaths due to birthing malformed lambs (table 4).

**Table 4:** Ewe mortality and assisted births by SBV category

Summary	SBV confirmed (n=59)	%	SBV suspected (n=82)	%	SBV not suspected (n=74)	%	P values
Breeding ewes that died during the lambing period (n)							0.108
0	16	28.6	25	32.5	25	41.0	
1–5	19	33.9	34	44.2	28	45.9	
6–10	7	12.5	8	10.4	3	4.9	
>10	14	25.0	10	13.0	5	8.2	
Ewes that died giving birth to a deformed lamb (n)							0.011
0	38	69.0	53	73.6	51	94.4	
1	4	7.3	7	9.7	1	1.9	
>1	13	23.6	12	16.7	2	3.7	
Ewes that gave birth to deformed lambs alone (n)							0.482
0	25	55.6	25	47.1	17	65.4	
1	7	15.6	13	24.5	5	19.2	
>1	13	28.9	15	28.3	4	15.4	
Ewes assisted by farmer because of a deformed lamb (n)							<0.001
0	8	20	12	21.8	18	66.7	
1	4	10	15	27.3	8	29.6	
>1	28	70	28	50.9	1	3.7	
Ewes assisted by vet because of a deformed lamb (n)							0.082
0	28	60.9	34	66.7	24	88.9	
1	10	21.7	10	19.6	3	11.1	
>1	8	17.4	7	13.7	0	0.0	
Caesarean sections because of deformed lamb (n)							0.008
0	31	67.4	37	75.5	24	100	
1	11	23.9	5	10.2	0	0	
>1	4	8.7	7	14.3	0	0	

Percentages may not add to 100 due to rounding.

SBV, Schmallenberg virus.

The difference in the number of caesarean sections between categories was significant (P=0.008), with 32.6 per cent (n=15) of respondents on SBV confirmed farms reporting one or more caesarean sections due to birthing a deformed lamb, 24.5 per cent (n=12) of respondents reporting the same on SBV suspected farms, compared with no caesareans due to birthing deformed lambs on SBV not suspected farms (table 4).

There was also a significant difference (P<0.001) between the number of respondents reporting farmer assistance of one or more ewes during lambing due to birthing a deformed lamb; 80 per cent (n=32) on SBV confirmed farms, 78.2 per cent (n=43) on SBV suspected farms and only 33.3 per cent (n=9) on SBV not suspected farms (table 4).

### Farmer perceived impacts

There was a significant difference between SBV category responses to the farmer perceived impact of SBV on the welfare of the flock, financial performance of the flock and farmers’ emotional wellbeing (P<0.001) (table 5).

**Table 5:** Perceived impact of SBV on the flocks’ welfare, the financial performance of flocks, the farmers’ emotional wellbeing and whether the respondent intends to give up sheep farming due to the impact of SBV this year by SBV category

Summary	SBV confirmed (n=59)	%	SBV suspected (n=82)	%	SBV not suspected (n=74)	%	P values
Impact of SBV on sheep flocks’ welfare	(58)		(81)		(67)		<0.001
No impact	11	19.0	31	38.3	60	90.0	
Strong positive impact	0	0.0	1	1.2	0	0.0	
Some positive impact	1	1.7	1	1.2	0	0.0	
Some negative impact	34	58.6	34	42.0	5	7.5	
Strong negative impact	12	20.7	14	17.3	2	3.0	
Impact of SBV on sheep flocks’ financial performance	(58)		(81)		(67)		<0.001
No impact	9	15.5	29	35.8	59	88.1	
Strong positive impact	0	0.0	0	0.0	0	0.0	
Some positive impact	1	1.7	1	1.2	1	1.5	
Some negative impact	31	53.4	36	44.4	7	10.4	
Strong negative impact	17	29.3	15	18.5	0	0.0	
Impact of SBV on farmers’ emotional wellbeing	(58)		(81)		(66)		<0.001
No impact	16	27.6	27	33.3	43	65.2	
Strong positive impact	0	0.0	0	0.0	0	0.0	
Some positive impact	1	1.7	0	0.0	1	1.5	
Some negative impact	23	39.7	37	45.7	21	31.8	
Strong negative impact	18	31.0	17	21.0	1	1.5	
Less likely to sheep farm next year because of SBV	(59)		(82)		(69)		0.014
	6	10.2	3	3.7	0	0.0	

SBV, Schmallenberg virus.

In total, 10.2 per cent of farmers on SBV confirmed farms and 3.7 per cent of farmers on SBV suspected farms reported that they were less likely to farm sheep again next year because of SBV. No farmer reported being less likely to farm sheep again next year because of SBV from SBV not suspected farms (table 5).

### Vaccination history

**Figure 5:**
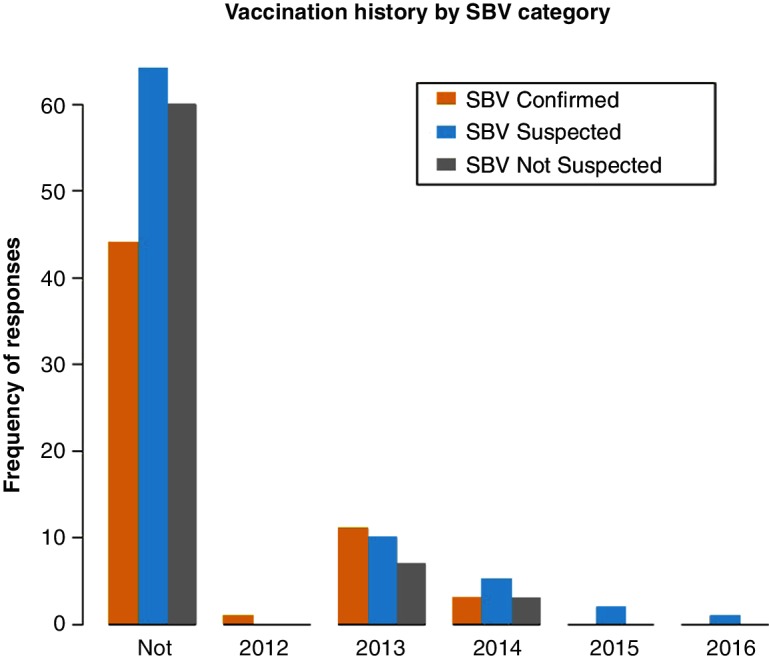
Frequency of reported vaccination history by SBV category. SBV, Schmallenberg virus.

There was no significant difference between SBV category and reported vaccination history (P=0.558). The majority of respondents had never previously vaccinated against SBV (79.6 per cent), with the most reported vaccinations against SBV occurring in 2013 (13.3 per cent) (figure 5).

### Current demand for vaccination

There was a small significant difference between the SBV categories and the price they would be willing to pay to vaccinate now against SBV (P=0.046). A higher proportion of respondents from SBV not suspected farms stated they would not vaccinate than respondents from SBV confirmed or SBV suspected farms (table 6). Roughly a third of respondents from SBV confirmed and SBV suspected farms stated they would consider vaccinating now if the vaccine costs less than £1, whereas just over a quarter of respondents from SBV not suspected farms would do the same.

**Table 6:** Respondents’ willingness to vaccinate against SBV at different prices for different SBV categories

Summary	SBV confirmed (n=59)	%	SBV suspected (n=81)	%	SBV not suspected (n=67)	%	P values
Would you consider vaccinating your sheep against Schmallenberg virus if it was available now?							0.046
No	11	18.6	13	15.9	21	31.3	
Yes, if it costs less than £1	19	32.2	29	35.8	18	26.9	
Yes, if it costs between £1 and £2	8	13.6	19	23.5	16	23.9	
Yes, if it costs between £2 and £3	12	20.3	13	16.0	5	7.5	
Yes, if it costs between £3 and £4	2	3.4	4	4.9	0	0.0	
Yes, if it costs between £4 and £5	7	11.9	3	3.7	7	10.4	

SBV, Schmallenberg virus.

## Discussion

This study has investigated the farm-level impact of SBV re-emergence on the 2016/2017 lambing season in the UK, allowing for comparisons to the initial 2011/2012 impact study by utilising the same methodology.^16^ Both this study and the original are limited by the reliance on farmer self-reporting of disease status rather than targeted testing. This survey focused on the impacts of the virus on lamb deformities, subsequent lambing problems and mortality events rather than monitoring for the effects of infection of adults outside of the susceptible period of gestation for deformities. SBV is not a notifiable disease in the UK, meaning that national-level systematic surveillance data are not available. It seems likely that most flocks were exposed to SBV in 2016/2017 as positive diagnostic submissions were reported across most areas of England, Wales and Southern Scotland.^22^ The respondents within this survey may be considered typical of the UK sheep farming community, as distribution of farm responses reflects the known density of sheep holdings in England and Wales, the respondents represented a range of farm sizes (from 3 to 3500 breeding ewes) and all major types of sheep farm were represented (hill, lowland, upland, pedigree and commercial farms).^25 26^ Therefore, in the absence of national disease prevalence data, the study can be considered a useful source of information on the farmer perceived, farm-level impact of SBV in the UK in 2016/2017 and allows comparison with the impact of the initial 2011/2012 outbreak.

As these survey data are farmer reported, there is the potential for reporting and recall bias. To address this possibility the categorisation of respondents into ‘SBV confirmed’, ‘SBV suspected’ and ‘SBV not suspected’ was highly conservative. Only respondents reporting cases on farm confirmed by the laboratory testing of a lamb were included in ‘SBV confirmed’, to ensure only RT-PCR confirmed cases were included in this grouping. All confirmed antibody serological testing of adult stock (ELISA and virus neutralisation test) was considered ‘suspected’, as it would be impossible to rule out historic antibodies, indicating exposure prior to the 2016/2017 outbreak. Only farmers answering ‘No’ and confirming no laboratory testing had been undertaken were designated ‘SBV not suspected’. False negatives in testing of lambs cannot be ruled out as this is a known issue with SBV testing.^27^ It is also very likely that many of the ‘SBV not suspected’ farms have had animals seroconvert (possibly outside of the period of pregnancy where malformations would be a risk) but as they had not seen the typical deformities and not tested for the virus were unaware of this. Similarly, false positives in the ‘suspected’ category may have occurred as not all congenital lamb malformations are due to SBV.

The significant difference observed between the SBV categories for lamb abnormalities provides justification for the current groupings, as if these were poorly differentiated then no observed differences in abnormalities would be seen. The fact that the SBV confirmed farms in general lambed earlier (and therefore would have had animals in the susceptible stages of pregnancy when the virus is thought to have circulated) when compared with the ‘not suspected’ group which generally lambed later and outside of this window also provides support for the current groupings.

In the present study the effects of SBV, reported by farmers, were increased neonatal lamb mortality, lambing mortality, dystocia and associated ewe deaths. In addition, farmers from SBV confirmed and suspected farms perceived that SBV had a significant negative impact on sheep welfare, the farms’ financial performance and their own emotional wellbeing. Farmers whose flocks were affected by SBV reported that they were less likely to farm again next year. Importantly, SBV confirmed and SBV suspected farms in this study typically described an earlier mating period than SBV not suspected farms, providing supportive evidence for the suggested period of disease re-emergence in the UK. The findings of the impact of the 2016/2017 SBV outbreak on sheep farms reported in the present study are largely comparable to the findings reported in the 2011/2012 outbreak, with the exception of ewe mortality. A comparative summary of results is presented in table 7 and is discussed below.

**Table 7:** A comparison table to directly compare the results of both studies for the studied factors

Factor	Harris and others’ 2011/2012 study^16^	This 2016/2017 study
Percentage of mated ewes that were barren*	No difference in median numbers between SBV confirmed (4), suspected (4.3) or not suspected (3.3) farms	No difference in median numbers between SBV confirmed (3.7), suspected (4.3) or not suspected (3.2) farms
Mating season	N/A	Difference between mating start date groups between SBV categories (figure 2)
Lambing season	No difference in median days between SBV confirmed (49.5), suspected (48.5) or not suspected (44.5) farms	Difference in median days between SBV confirmed (64.5), suspected (52.0) and not suspected (40.0) farms
Lambing percentage*	No difference in median numbers between SBV confirmed (169.1%), suspected (166.7%) or not suspected (164.2%) farms	No difference in median numbers between SBV confirmed (174.3%), suspected (173.0%) or not suspected (166.7%) farms
Scanning percentage	NA	No difference in median numbers between SBV confirmed (175.0%), suspected (172.5%) or not suspected (176.0%) farms
Lamb mortality*	Higher mortality SBV confirmed (10.4%), suspected (7.0%) than not suspected (5.3%)	Higher mortality SBV confirmed (9.1%), suspected (7.6%) than not suspected (5.7%)
Lambing mortality*	Higher mortality SBV confirmed (18.2%), suspected (11.3%) than not suspected (8.6%)	Higher mortality SBV confirmed (15.2%), suspected (12.7%) than not suspected (8.4%)
Number of breeding ewes that died during the lambing period	More ewes dying on SBV confirmed (66.7%), SBV suspected (67.1%) than not suspected (54.5%) farms	No difference SBV confirmed (71.4%), suspected (67.5%) or not suspected (59%) farms
Number of ewes died giving birth to deformed lambs*	More dying on SBV confirmed (36.9%), suspected 16.8%) than not suspected (7.2%) farms	More dying on SBV confirmed (30.9%), suspected (28.4%) than not suspected (5.6%) farms
Number of ewes that gave birth to deformed lambs alone	NA	No difference between SBV confirmed (44.4%), suspected (52.9%) or not suspected (34.6%) farms
Number of ewes assisted by farmer because of a deformed lamb	NA	More ewes assisted on SBV confirmed farms (80%), suspected (78.2%) than not suspected (33.3%) farms
Number of ewes assisted by vet because of a deformed lamb	More ewes assisted on SBV confirmed farms (35.8%), suspected (19.5%) than not suspected (4.8%) farms	No difference between SBV confirmed (39.1%), suspected (33.3%) or not suspected (11.1%) farms
Number of caesarean sections because of deformed lambs*	More caesareans on SBV confirmed (12.3%), suspected (11%) than not suspected (1.6%) farms	More caesareans on SBV confirmed (32.6%), suspected (24.5%) than not suspected (0%) farms
Farmer perceived impact of SBV on sheep welfare†	Higher impact (4 or 5) on SBV confirmed (36.8%), suspected (17.8%) than not suspected (0.5%) farms	Higher negative impact on SBV confirmed (79.3%), suspected (59.3%) than not suspected (10.5%) farms
Farmer perceived impact of SBV on financial performance†	Higher impact (4 or 5) on SBV confirmed (32.8%), suspected (20.1%) than not suspected (2.3%) farms	Higher negative impact on SBV confirmed (82.7%), suspected (62.9%) than not suspected (10.4%) farms
Farmer perceived impact of SBV on farmers’ emotional wellbeing†	Higher impact (4 or 5) on SBV confirmed (49.3%), suspected (25.6%) than not suspected (6.5%) farms	Higher negative impact on SBV confirmed (70.7%), suspected (61.7%) than not suspected (33.3%) farms
Less likely to sheep farm next year because of SBV	No difference between SBV confirmed (5.7%), suspected (5.9%) than not suspected (1.8%) farms	Higher numbers less likely to sheep farm next year on SBV confirmed (10.2%), suspected (3.7%) than not suspected (0%) farms

*Similar findings reported in both studies.

†Methodology differs so not directly comparable. Differences were at the P<0.05 significance. Data summarised for 2011/2012 outbreak.^16^

NA, not applicable; SBV, Schmallenberg virus.

Mating start dates and lambing season duration were found to be significantly different between the SBV categories. Importantly, SBV confirmed and SBV suspected farms typically started mating in July/August (61 and 44 per cent, respectively), whereas the majority of SBV not suspected farms (69 per cent) reported mating in September/October; this would put the vulnerable period of gestation for fetal deformities (approximately days 28–56 of pregnancy) for these later mated flocks largely outside of the autumn activity peak of the SBV *Culicoides* vector species.^18 28^ As SBV was determined to have circulated in *Culicoides* in August 2016 in Belgium,^29^ and due to confirmed SBV malformations in lambs in south England beginning in December, peaking across England in January and February 2017,^22^ it is likely that SBV circulated widely in England in September/October 2016.^22 29^ This is further supported by unpublished data from the University of Nottingham flock, demonstrating seroconversion to SBV and viral RNA in October 2016. This timing of circulation agrees with the results of this study: that those worst affected by fetal malformations were flocks mating in August/September 2016.

Questions have been raised regarding the impact of SBV on early reproductive losses in sheep flocks.^17^ However, in the present study there was no difference in the reported barren ewe rate between SBV categories, nor was a difference reported in the previous 2011/2012 study. Indeed, the reported median barren rates in this study (3.7 per cent) were very similar to those reported for the 2011/2012 outbreak (4 per cent)^16^ and although these barren ewe rates are higher than industry guidelines,^30^ they appear to be typical of UK sheep flocks.^25 31^ A study on SBV infection and early ewe reproductive performance in the Netherlands also failed to find associations.^10^

The lamb mortality and lambing mortality were significantly higher on SBV confirmed and SBV suspected farms, with almost double the median lamb mortality and lambing mortality on SBV confirmed farms (median 15.2 and 9.1 per cent, respectively) compared with SBV not suspected farms (median 8.4 and 5.7 per cent, respectively). These results were very similar to those reported in the UK during 2011/2012 lambing and in European studies.^10 11 13 16 17^ The lamb mortality on SBV not suspected farms reported here was comparable to industry figures and studies of lamb mortality in UK flocks, prior to the 2011 SBV outbreak.^25 32^

Increased lamb mortality observed on SBV affected farms is likely an effect of the associated congenital malformations and behavioural abnormalities on the lamb’s ability to adapt to postnatal life. Several congenital malformations were associated with SBV infection in the present study. Twisted limbs were the most frequently reported malformation of lambs on SBV confirmed and SBV suspected farms, followed by curved backs and deformed heads. This agrees with the previous UK experience of SBV. Several farmers reported eye deformities, including blindness; previously reported by farmers in the 2012/2013 study and in an SBV clinical investigation of a calf.^16 33^ Some farms in the not suspected category did have lambs with deformities that could be consistent with SBV, though at a very much lower rate than the SBV farms. Some of these may have been assumed to be due to teratogens like Border disease that farmers are more familiar with in the UK and that can produce similar deformities. This highlights the need for testing as congenital malformations in lambs are not exclusive to SBV infection and can be the result of a wide range of teratogenic, genetic or nutritional factors.^34^ This also helps to prevent the assumption that all malformations are SBV, ensuring the cause is properly investigated and diagnosed. The data from such testing also provide passive surveillance, which can act as an alert for an increase in viral circulation or reduction in national flock immunity to SBV.

Overall there was no difference in ewe mortality across SBV categories in the present study. Although unsurprisingly, both ewe mortality associated with birthing malformed lambs and the number of assisted births (both by the farmer and by caesarean) were greater on SBV affected farms. More caesarean sections were also reported on SBV confirmed and SBV suspected farms in this study (33 and 25 per cent, respectively) than reported during the initial 2011/2012 outbreak (12 and 11 per cent, respectively).^16^ Certainly the delivery of malformed lambs presents increased risk to ewe health, although there was no evidence for an impact on overall ewe mortality as previously reported.^16^ This could be due to improved farmer/veterinary awareness of the risk to ewe health, and therefore earlier appropriate obstetrical intervention.

As expected the perceived welfare, economic and emotional impact of SBV to farmers was generally high on SBV confirmed and suspected farms, and low on SBV not suspected farms. The greatest reported negative impact was on farmers’ emotional wellbeing. As SBV is no longer novel, the distressing nature of the associated malformations is well known among farming communities. It is likely that this awareness, along with potential previous experience of the disease, is likely to have contributed to the high proportion of negative emotional impact of SBV reported, even on unaffected farms. A total of 4.3 per cent respondents stated they were less likely to sheep farm next year because of SBV. This is comparable to the proportion of respondents stating the same after the previous SBV outbreak (3.7 per cent).^16^

Although the data do not provide a complete description of the 2016/2017 UK outbreak, it is interesting that the geographic distribution of SBV confirmed farms in this sample did deviate from the previous 2011/2012 outbreak distribution. In 2011/2012 the outbreak began in south-east England and rapidly spread in a north-westerly direction covering the majority of England and Wales to the Scottish Border.^35^ Here, despite the survey being distributed nationally, the majority of SBV positive farms were in the west of England and Wales. Perhaps the difference in response distribution between the surveys reflects a different route or timing of disease introduction between the 2011/2012 and 2016/2017 outbreaks.^22^

Vaccination history against SBV was also explored. Only one-fifth (20.2 per cent) of respondents stated they had vaccinated against SBV previously; however, over 78 per cent of respondents stated they would consider paying to vaccinate against SBV if the vaccine was available. Interestingly, more respondents from SBV confirmed farms stated they had vaccinated in 2013 than SBV suspected or SBV not suspected farms. It may be the farm management practices place them at higher risk of SBV and therefore they are more conscious of disease risk and more likely to vaccinate.

Under-reporting of SBV cases is recognised as an issue for measuring the impact of the disease on populations.^14^ The number of suspected cases in this study that were not sent off for testing, in areas where confirmed positives existed, also highlights the potential extent of under-reporting. Surveys such as this represent the only way to estimate the potential extent of impact on farm of non-notifiable diseases. Although many farmers would send off a single suspected case for testing, it would be unusual, due to the upfront and time costs of testing during an extremely busy period for sheep farms (lambing), to send off all suspected cases on farm for confirmatory testing.^14^ Additionally, as infection of adult ruminants is typically asymptomatic it is likely that infection occurred on many unsuspecting farms, just outside of the vulnerable period of gestation for deformities. This may account for why no significant differences were observed between SBV categories and early oestrus factors such as barren rates, lambing percentages and scanning percentages.

The results of this survey clearly demonstrate an impact of SBV on the 2016/2017 lambing season, comparable to that reported for the 2011/2012 lambing season.^16^ If SBV transmission continues to be cyclical in nature, the associated animal welfare and subsequent economic costs to the UK sheep farming industry will continue every few years if intervention is not taken. Controlling the *Culicoides* vector has so far been unfeasible, subsequently the importance of timely vaccination (if available) or coinciding mating with colder periods will continue to be necessary to reduce the impact of future SBV outbreaks. National surveillance programmes, particularly collaborative surveillance programmes with European member states, are increasingly important for the application of timely vaccination programmes; however, vaccination production ceases when demand is low. Future studies should aim to address this cyclical epidemiology, particularly identifying where the virus persists between outbreaks to allow the development of predictive early-warning models.
